# The main decision-making competence for willingness-to-pay towards COVID-19 vaccination: a family-based study in Taizhou, China

**DOI:** 10.1080/07853890.2022.2114606

**Published:** 2022-08-25

**Authors:** Chengwen Luo, Mei-Xian Zhang, Eva Jiang, Mindan Jin, Tao-Hsin Tung, Jian-Sheng Zhu

**Affiliations:** a Evidence-Based Medicine Center, Taizhou Hospital of Zhejiang Province, Wenzhou Medical University, Linhai, Zhejiang, China; b Gucheng Street Community Health Service Center, Linhai, Zhejiang, China; c Department of Infectious Diseases, Taizhou Hospital of Zhejiang Province, Wenzhou Medical University, Linhai, Zhejiang, China

**Keywords:** Decision making, COVID-19 vaccination, willingness to pay, community, China

## Abstract

**Purpose:**

This research aimed to explore individuals’ willingness to pay (WTP) and studied the role of family decision makers in WTP for COVID-19 vaccines.

**Methods:**

A self-administered online questionnaire evaluating the willingness of community residents to pay for booster vaccination of COVID-19 vaccine was conducted among families in a community in Taizhou, China. The logistic regression model was performed to identify the factors associated with WTP for the COVID-19 vaccines, and all data were analysed by R software, version 4.1.0.

**Results:**

44.2% and 43.7% of 824 community residents were willing to pay for the first two doses and the booster dose of the COVID-19 vaccine, respectively. Decision-makers were more willing to pay for both the first two doses and the boost dose of the COVID-19 vaccines, with OR (95%CI) being 1.75 (1.25–2.47) and 1.89 (1.34–2.67), respectively. Besides, participants’ WTP for COVID-19 vaccines were also associated with their occupation and monthly household income.

**Conclusion:**

This study found that family decision-makers were more willing to pay for both the first two doses and the booster dose of COVID-19 vaccines in Taizhou, China. To improve the WTP for COVID-19 vaccines, public policy programs need to conduct a comprehensive cost-benefit analysis and focus on the role of family decision makers in vaccination.

**Key Messages**
- A study evaluating the willingness of community residents to pay for booster vaccination of COVID-19 vaccine was conducted among families in a community in Taizhou, China.
- Family decision-makers were more willing to pay for both the first two doses and the booster dose of COVID-19 vaccines.
- To improve the WTP for COVID-19 vaccines, public policy programs need to conduct a comprehensive cost-benefit analysis and focus on the role of family decision-makers in vaccination.

## 1. Introduction

A new coronavirus (SARS-CoV-2) was announced as a public health crisis by WHO in January 2020, which has caused severe acute respiratory syndrome symptoms. The disease was later named coronavirus disease 2019 (COVID-19) and declared a pandemic in March 2020 [1,2]. The pandemic has appreciably influenced morbidity and mortality and has been a major international threat [3,4]. A large number of studies have been carried out to seek effective prevention methods against the pandemic [5–7]. As part of the response to the pandemic, the COVID-19 vaccines are a potentially effective means of reducing spread rates and subsequent infections. The mass immunisation programme has progressed well since its launch in December 2020, with at least five different COVID-19 vaccines (i.e. one recombinant protein vaccine, one adenovirus vector vaccine, and three inactivated vaccines) approved for emergency use as of March 2021 in China. In late September 2021, it was reported that China was planning a COVID-19 vaccine booster so that people who had completed two-dose immunisation six months before could obtain protection.

To contain and prevent the current COVID-19 pandemic and future outbreaks, vaccination strategies aimed at achieving high vaccination coverage should address vaccination availability and financial affordability. Lessons learned from the 2009 H1N1 pandemic influenza underscore that inadequate financial affordability and timely distribution of adequate vaccines can lead to failure to prepare for and respond to pandemics, particularly in low- and middle-income countries [8]. This illustrated that appropriate pricing and financing mechanisms were important for pandemic vaccination. To date, vaccines are distributed through the public sector in China, nevertheless, they may be available in the private market in the future. Although the COVID-19 vaccine has shown its safety and efficacy, it remains unclear whether people will accept and purchase the vaccine. Hence, it is of great importance to assess the willingness to pay (WTP) for the COVID-19 vaccines.

WTP is defined as the maximum amount of money that people are willing to pay for a project and is a conditional assessment consisting of a hypothetical survey that directly asks the amount an individual would be willing to pay [9–11]. In China, COVID-19 vaccines are currently available free of charge, however, based on the conditional valuation approach, a hypothetical scenario for individuals with ongoing outbreaks is provided, in which the cost of the vaccine is paid for by the individual [12]. Information about people’s WTP for a hypothetical vaccine against the virus could aid future price-setting discussions and contribute to decision-making to inform potential pricing for a hypothetical COVID-19 vaccine.

Recent decades have seen many studies focussed on assessing the WTP of vaccines [13–16]. Identifying the factors associated with WTP for COVID-19 vaccines is important for governments and organisations to develop a well-designed intervention program for use in the population. Studies have shown that factors, such as socio-demographic characteristics, beliefs, and pre-existing attitudes, were associated with WTP [13–16]. People usually take costs and benefits related to COVID-19 into consideration when deciding to get vaccinated. Costs consist of opportunity and monetary costs and any discomfort resulting from such measures, while benefits depend on the severity of illness at the time a person becomes infected, the likelihood of infection, as well as the effectiveness of prevention measures. Family decision-makers means the people who make decisions in their family and the decisions of the family decision-makers can affect the choices of the entire family. However, there is the paucity of research on the role of family decision-makers on WTP for COVID-19 vaccines.

To fill the gap in WTP for the COVID-19 vaccines in China, this study aims to investigate the relationship between decision makers for COVID-19 vaccination and the willingness to pay for COVID-19 vaccines. The study of acceptance and the willingness for the COVID-19 vaccine are essential to assess the feasibility of implementing a vaccination program once the vaccine is available, as well as provide insights for future pricing considerations and demand projections.

## 2. Method

### 2.1. Study design

We commenced a cross-sectional community-based online survey through the WeChat-incorporated Wen-Juan-Xing platform, which is used reachable to a large population in China. Our target population was families in a community in Taizhou, China. The participants voluntarily answered the self-administered questionnaire by scanning the Quick Response (QR) code on their mobile phones from September 1st to 15th, 2021. After quality control, 402 households (402/1002) and a total of 824 interviewees with valid data were included in this study.

This study was approved by the Ethics Committee of Taizhou Hospital of Zhejiang Province (approval number: K20210705) in China. All programs were carried out according to the standards of our ethics committee and adhered to the tenets of the Declaration of Helsinki. Oral rather than written consent was used and the information of respondents was kept anonymous. All respondents’ information was anonymous.

### 2.2. Questionnaires

The questionnaire we designed was based on previous research on assessing willingness to pay for vaccines [17–19]. To ensure that the formal questionnaire was comprehensive, scientific, and unambiguous, we conducted pilot interviews to test and validate the questionnaire. Interviewers participated in a training course on the questionnaire content. In the early stage, we conducted a pilot test on 30 volunteers to confirm whether there were omissions or unanswerable parts in the questionnaire.

The questionnaire included the following information mainly based on our previous study about parents’ willingness to pay for their children [19]. First, we collected information on basic characteristics, including sex, age, occupation, education level, and monthly household income. Family monthly income was measured by asking the question, “What is your monthly household income?” (five items: <5000, 5000–9999, 10000–19999, 20000–29999, or ≥30000 Chinese Yuan (CNY)). Second, the family decision maker was measured by asking the question, “Are you the primary decision maker for your family members’ COVID-19 vaccination?” (two items: yes; no). Third, allergic history was measured by asking the question, “Any previous history of food or drug allergies?” (two items: yes; no). Underlying disease was measured by asking the question, “Do you suffer from any of the following chronic diseases, including hypertension, diabetes, chronic respiratory disease, cardiovascular disease, chronic liver disease, chronic kidney disease, and cancer?”. Allergic reactions to other vaccines was measured by the question, “Any previous allergic reactions to other vaccines?”. Fourth, the willing-to-pay for the first two doses of the COVID-19 vaccine was measured by a question asking whether respondents were willing to receive the booster injection if they have to pay for it. The amount of payment was measured through the question asked what the maximum price they were willing to pay for the vaccine. The 6 response categories were as follows: 1) less than 100; 2) 100–199; 3) 200–299; 4) 300–399; 5) 400–499; and 6) more than 500 (Chinese Yuan (CNY)). Fifth, similar questions regarding willingness to pay for the booster dose of the COVID-19 vaccine were also asked.

### 2.3. Statistical analysis

The main outcomes of the study were willing to pay for the first two doses of the COVID-19 vaccine and the booster dose. Counts and percentages were shown for categorised variables. We used the Chi-Square test to compare the differences between the willing-to-pay and unwilling-to-pay groups. The logistic regression model was adopted to identify variables associated with WTP for COVID-19 vaccines. All data were analysed by R software, version 4.1.0 (R Project for Statistical Computing).

## 3. Result

### 3.1. Sample characteristics

We collected a total of 824 respondents with valid data from September 1st to 15th, 2021 in Taizhou, Zhejiang, China. The demographics of the participants were summarised in Table 1. A total of 369 (44.8%) participants were the primary decision makers for the COVID-19 vaccination of family members. Among the survey respondents, half were male. Their mean age was 41.9(±17.0) years old. 43.9% of the participants were over 45 years old. Besides, 21.1% of respondents held a junior college degree or above. A total of 21.7% of participants were blue-collar or farmers. More than half of the participants had monthly household incomes ranging from 5000 to 9999 yuan. Most respondents had no allergic history, underlying disease, or any allergic reactions to other vaccines. Of the 824 study participants, 364 (44.2%) individuals were willing to pay for the first two doses of the COVID-19 vaccines, and 360 (43.7%) were willing to pay for the booster dose.

**Table 1.** Descriptive statistics (*N* = 824).

Characteristics	*n* (%)	Willingness to pay for vaccine of the first two doses (*n* = 364)	Willingness to pay for vaccine of the booster dose (*n* = 360)
Decision maker			
Yes	369 (44.8)	179 (49.2)	178 (49.4)
No	455 (55.2)	185 (50.8)	182 (50.6)
Age (years)			
<30	215 (26.1)	99 (27.2)	98 (27.2)
30–44	247 (30.0)	114 (31.3)	108 (30.0)
45–59	257 (31.2)	115 (31.6)	117 (32.5)
≥60	105 (12.7)	36 (9.9)	37 (10.3)
Sex			
Male	412 (50.0)	181 (49.7)	173 (48.1)
Female	412 (50.0)	183 (50.3)	187 (51.9)
Education			
Primary school or below	227 (27.6)	96 (26.4)	95 (26.4)
Middle school	220 (26.7)	90 (24.7)	91 (25.3)
High school	203 (24.6)	104 (28.6)	101 (28.1)
Junior college	80 (9.7)	32 (8.8)	35 (9.7)
Bachelor degree or above	94 (11.4)	42 (11.5)	38 (10.5)
Occupation			
Student	157 (19.1)	61 (16.8)	61 (16.9)
Blue-collar/farmer	179 (21.7)	53 (14.6)	56 (15.6)
White-collar	20 (2.4)	15 (4.1)	15 (4.2)
Others	468 (56.8)	235 (64.5)	228 (63.3)
Monthly household income (Chinese Yuan)			
<5000	226 (27.4)	92 (25.2)	94 (26.1)
5000–9999	464 (56.3)	180 (49.5)	176 (48.9)
10000–19999	130 (15.8)	91 (25.0)	89 (24.7)
≥20000	4 (0.5)	1 (0.3)	1 (0.3)
Allergic history			
No	807 (97.9)	358 (98.4)	352 (97.8)
Yes	17 (2.1)	6 (1.6)	8 (2.2)
Underlying disease			
No	751 (91.1)	332 (91.2)	325 (90.3)
Yes	73 (8.9)	32 (8.8)	35 (9.7)
Allergic reactions to other vaccines			
No	808 (98.1)	354 (97.3)	351 (97.5)
Yes	16 (1.9)	10 (2.7)	9 (2.5)

### 3.2. Willingness to pay for vaccines regarding to COVID-19

**Figure 1.**
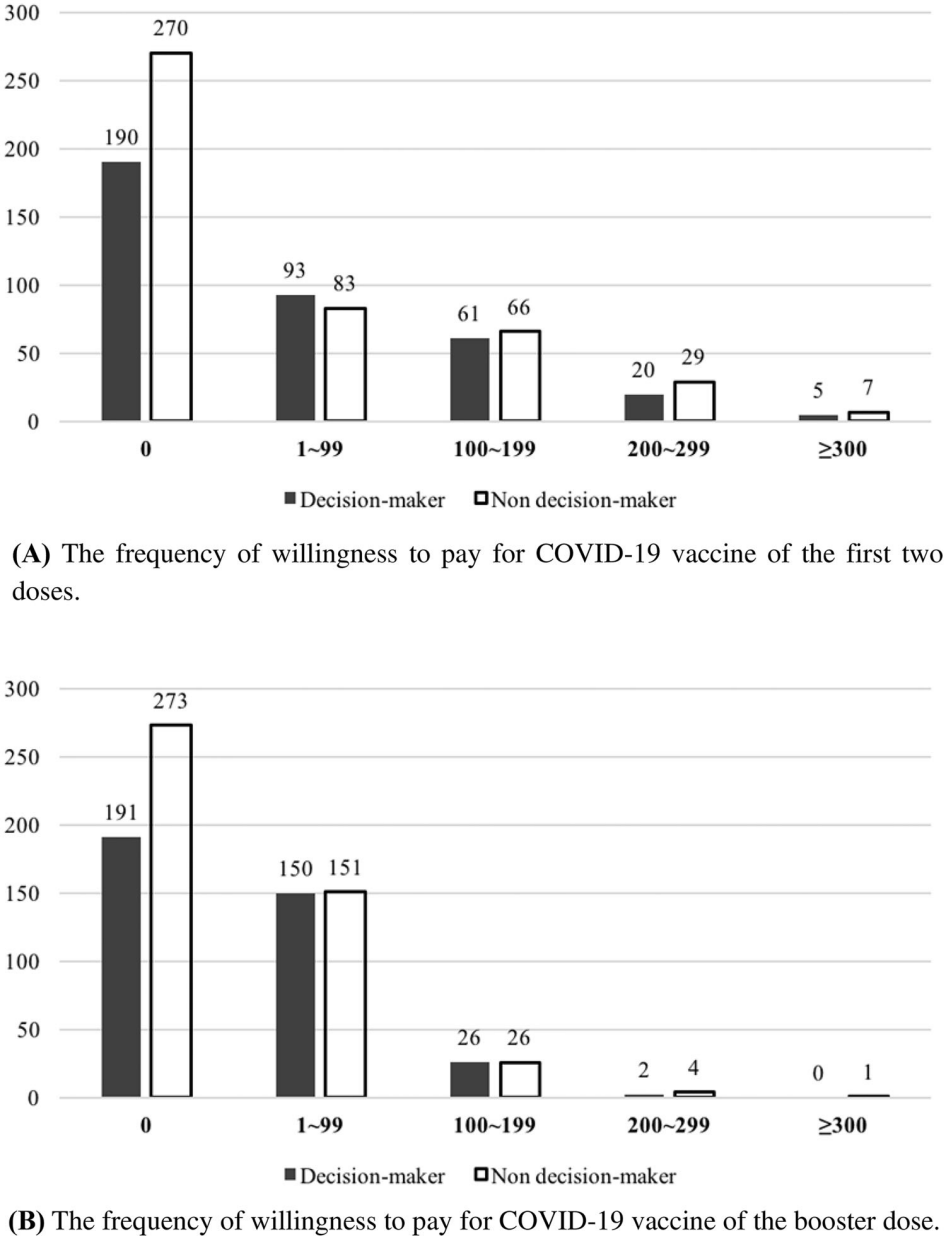
The frequency of amount willing to pay for COVID-19 vaccines. For (A), the chi-square value for the comparison of the decision-maker group and non-decision-maker group was 7.77, and there was no significant difference between the two groups (*P*-value = .10). For (B), the chi-square value for the comparison of the decision-maker group and non-decision-maker group was 7.26, and there was no significant difference between the two groups (*P*-value = .12).

Figure 1 reported the frequency of participants’ willingness to pay for a vaccine against COVID-19. There were 364 (44.2%) participants who were willing to pay for the first two doses of the COVID-19 vaccine. Of these 460 participants who were unwilling to pay, 58.7% were not the decision-makers in the vaccination. Among those who were willing to pay, there were 176 (48.4%) participants were willing to pay CNY 1–99 for the first two doses of the COVID-19 vaccine, and 52.8% were decision makers for COVID-19 vaccination. 34.9% of individuals were willing to pay CNY 100–199 for the first two doses. Only 16.7% of participates were willing to pay more than CNY 200. The chi-square value for the comparison of the decision-maker group and non-decision-maker group was 7.77, and there was no significant difference between the two groups (*P*-value = .10).

For the booster dose of the COVID-19 vaccine, 360 (43.7%) individuals were willing to pay. Among the 464 individuals who were unwilling to pay, 58.8% were not the decision-makers in the vaccination. Compared to the first two doses of the COVID-19 vaccine, 301 (83.6%) were willing to pay less than CNY 100 for the booster dose. The chi-square value for the comparison of the decision-maker group and non-decision-maker group was 7.26, and there was no significant difference between the two groups (*P*-value = .12).

### 3.3. Factors associated with willingness to pay for COVID-19 vaccine

Table 2 summarised the results of the univariate analysis. Willingness to pay for the first two doses of the COVID-19 vaccines was related to participants’ role in COVID-19 vaccination (i.e. decision maker or non-decision maker) ($\chi^{2}$ = 4.778, *P*-value = .029), occupation ($\chi^{2}$ = 31.830, *P*-value < .001), and monthly household income ($\chi^{2}$ = 42.305, *P*-value < .001). Similarly, willingness to pay for the booster dose of the COVID-19 vaccine was related to participants’ role in COVID-19 vaccination ($\chi^{2}$ = 5.291, *P*-value = .021), occupation ($\chi^{2}$ = 25.468, *P*-value < .001), and monthly household income ($\chi^{2}$ = 39.652, *P*-value < .001).

**Table 2.** Univariate analysis of factors associated with willingness to pay for the COVID-19 vaccines.

Characteristics	*n*	The first two doses			The booster dose		
		%	$\chi^{2}$	*P*	%	$\chi^{2}$	*P*
Total	824	44.2			43.7		
Decision maker			4.778	.029		5.291	.021
Yes	369	48.5			48.2		
No	455	40.7			40.0		
Age (years)			4.896	.180		3.713	.294
<30	215	46.0			45.6		
30–44	247	46.2			43.7		
45–59	257	44.7			45.5		
≥60	105	34.3			35.2		
Sex			0.005	.944		0.834	.361
Male	412	43.9			42.0		
Female	412	44.4			45.4		
Education			5.953	.203		4.238	.375
Primary school or below	227	42.3			41.9		
Middle school	220	40.9			41.4		
High school	203	51.2			49.8		
Junior college	80	40.0			43.8		
Bachelor degree or above	94	44.7			40.4		
Occupation			31.830	<0.001		25.468	<0.001
Student	157	38.9			38.9		
Blue-collar/farmer	179	29.6			31.3		
White-collar	20	75.0			75.0		
Others	468	50.2			48.7		
Monthly household income (Chinese Yuan)			42.305	<0.001		39.652	<0.001
<5000	226	40.7			41.6		
5000–9999	464	38.8			37.9		
10000–19999	130	70.0			68.5		
≥20000	4	25.0			25.0		
Allergic history			0.248	.618		0.001	.971
No	807	44.4			43.6		
Yes	17	35.3			47.1		
Underlying disease			0.000	1		0.415	.519
No	751	44.2			43.3		
Yes	73	43.8			47.9		
Allergic reactions to other vaccines			1.528	.216		0.590	.442
No	808	43.8			43.4		
Yes	16	62.5			56.3		

The effects of independent factors on willingness to pay for the COVID-19 vaccines were examined *via* a logistic regression model. As presented in Table 3, family decision-makers were a significant factor for WTP for both the first two doses and the booster dose of the COVID-19 vaccines, with an odds ratio (OR) (95%CI) be 1.75 (1.25–2.47) and 1.89 (1.34–2.67), respectively. Besides, compared with students, blue-collars or farmers had a lower willingness to pay for the COVID-19 vaccines (0.53 (0.32–0.88)), while white-collars were more willing to pay (3.18 (1.10–10.58)). Furthermore, monthly household income (10000–19999 vs. <5000, OR = 2.21, 95%CI: 1.34–3.67) was significantly associated with their willingness to pay for the first two doses of the COVID-19 vaccines. Similar results could be obtained for willingness to pay for the booster dose. Compared with students, blue-collars or farmers had less willingness to pay (0.56 (0.34–0.93)), while white-collars were more willing to pay (3.22 (1.12–10.70)). Monthly household income (10000–19999 vs. <5000, OR = 2.08, 95%CI: 1.27–3.45) was significantly associated with their willingness to pay for the booster dose of the COVID-19 vaccine.

**Table 3.** Regression results.

Characteristics	The first two doses			The booster dose		
	OR	95% CI	*P*	OR	95% CI	*P*
Decision maker						
No (Ref)						
Yes	1.60	1.17–2.20	.003	1.63	1.19–2.24	.002
Occupation						
Student (Ref)						
Blue-collar/ Farmer	0.53	0.32–0.88	.014	0.56	0.34–0.93	.024
White-collar	3.18	1.10–10.58	.041	3.22	1.12–10.70	.039
Others	1.29	0.86–1.94	.218	1.21	0.81–1.83	.353
Monthly household income (Chinese Yuan)						
<5000 (Ref)						
5000–9999	0.65	0.45–0.93	.019	0.63	0.44–0.89	.010
10000–19999	2.21	1.34–3.67	.002	2.08	1.27–3.45	.004
≥20000	0.31	0.01–2.51	.314	0.31	0.02–2.54	.320

## 4. Discussion

The COVID-19 pandemic has dramatically affected the lives of people worldwide and caused significant disease and economic burdens in the world. Vaccination was considered a cost-effective way to control and prevent infectious diseases. Considering that the outbreak might have a continued influence on human beings, we may have to be prepared for ongoing vaccinations. Although vaccines are currently free in China, there is a possibility that the public will have to pay for them in the future due to the uncertainty of the duration of the pandemic. For the government and social organisations, there is a need to understand whether the general public will still be willing to receive vaccines if they have to pay for them, and what the public will accept as the pricing of vaccines.

This research explored individuals’ WTP and studied the role of decision-maker in willingness to pay for COVID-19 vaccines. We found that 44.2% and 43.7% of 824 community residents were willing to pay for the first two doses and the booster dose of the COVID-19 vaccine, respectively. Decision-makers were more willing to pay for both the first two doses and the boost dose of the COVID-19 vaccines, with OR (95%CI) being 1.75 (1.25–2.47) and 1.89 (1.34–2.67), respectively. Besides, the results showed that participants’ willingness to pay for COVID-19 vaccines was also associated with their occupation and monthly household income. Monthly household income affected participants’ WTP, which was similar to previous studies [20–22]. Participants who were white-collars, such as respondents with professional and managerial occupations had a higher WTP over the lower amount [23].

We also analysed different payments for the COVID-19 vaccines and found that among those who were willing to pay for the first two doses of the COVID-19 vaccine, participants were willing to pay CNY 99 or less (48.4%), CNY 100–199 (34.9%), and more than CNY 200 (16.7%). There was no significant difference between the decision-maker group and the non-decision-maker group. Similar findings were found in the booster dose. A previous study showed that participants with a mental disorder had higher WTP than healthy controls (64.5% vs. 38.1%) in Chongqing, China [24]. Among the healthy controls, non-healthcare workers, health insurance, living with children, and internalised stigma were significant factors for WTP. A survey conducted in Vietnam reported that 82.6% of 651 pregnant women were willing to pay for the COVID-19 vaccine and the mean amount of WTP was USD 15.2 (±27.4) [25]. The average WTP was reported to be about MYR 134.0 (USD 30.6) in Malaysia and USD 184.7 in Chile [23,26]. In Indonesia, the average WTP for the Covid-19 vaccine was USD 57.20 [27]. A more general study in ten low-middle-income countries (LMICs) in Asia, Africa, and South America showed that the average WTP is USD 87.9 [28].

The decision-maker role is rarely enacted in isolation. Multiple family members, for example, usually participated in the decision-making process of making choices when there was a need to solve problems, and family members expected consensus in the decision [29,30]. Given the interdependence of families and the value of family participation in the decision-making process, expectations and behaviours about the role of the decision-maker may emerge and form in interactions with others in the family. Therefore, in future vaccine campaigns, we could focus more attention on family decision-makers, which might achieve better results. Besides, evidence from studies indicated that cost-effectiveness plays a major role in decision-making [31–33], which might explain the reason that there was no significant difference in the amount paid for vaccines between the decision-maker and non-decision-maker groups. In addition, a number of variables identified in this research were associated with participants’ WTP. Household income was found to be a significant factor for WTP, which was similar to previous studies that economic variables had an impact on WTP [27,34–36]. This might reflect a direct association between WTP and the ability to pay, or an indirect association in that people with higher incomes may be more knowledgeable about the benefit of vaccination. As for the research on the willingness to pay for COVID-19 vaccine of different occupational groups, previous studies have shown that individuals with managerial occupations had higher marginal WTP for the vaccines and farmers were less willing to pay for the vaccine than white-collar workers [23,25].

There are several limitations in this research that need to be further studied. First, the sample may not be fully representative, since we only considered residents in a community. Besides, there might be differences between different communities. In addition, the research population was selected on a voluntary basis, which not only would potentially introduce selection bias but also the Hawthorne effect was inevitable. Hence, in order to further identify the role of decision-making in taking COVID-19 vaccines, the generalisation and external validity should be further studied. Second, the online data collection method was a limitation of this study, which could potentially lead to over-reporting or lower-reporting the willingness to pay of the COVID-19 vaccines. Third, in this study, we used only one question to ask whether the respondent is the decision-maker in his/her family. It is possible that their roles are similar to other family members in this study since they merely represent themselves in determining the WTP of the COVID-19 vaccine. In future studies, we need to determine the main decision maker more clearly. The last, this research was investigated in only a cross-sectional survey, which is difficult to reflect the long-term exposure to variables. Hence, in future studies, it is important to conduct longitudinal research or a larger sample size.

## 5. Conclusion

This study investigated the potential factors related to willingness to pay for COVID-19 vaccines and found that the main decision-maker in his/her family was more willing to pay for both the first two doses and the booster dose of COVID-19 vaccines in Taizhou, China. To improve the WTP for COVID-19 vaccines, public policy programs need to conduct a comprehensive cost-benefit analysis and focus on the role of family decision makers in vaccination.

## Data Availability

The datasets generated and/or analysed during the current study are available from the corresponding author on request.
